# Our Book Table

**Published:** 1885-02

**Authors:** 


### OUR BOOK TABLE.

A Text-Book of Hygiene. *A Comprehensive Treatise on the Principles and practice of Preventive Medicine from an American Standpoint.* By George H. Rohe, M. D., Professor Hygiene, College of Physicians and Surgeons, Baltimore; Member American Public Health Association; of the Medical and Chirurgical Faculty of Maryland, etc., etc. Baltimore: Thomas & Evans, 1885, Octavo pp. 324.

Within the past decade hygienic science has made important strides, and a constantly increasing interest is manifested in preventive medicine. If it be better to avoid disease entirely, rather than to secure convalescence after a perhaps lingering illness, then is hygiene one of the most important branches of medical study. With the advance in knowledge concerning all zymotic and septic diseases, comes perception of the fact that it is to prophylactic medicine that we must chiefly look for relief from a large class of the hitherto most fatal disorders to which man is subject. Especially is it important that the physician who places any faith whatever in the bacterian origin of certain diseases, should become thoroughly conversant with the conditions which most favor the growth of micro-organisms. And even those illnesses that are not supposed to have any connection with the multiplication of microbes, are yet largely dependent upon hygienic conditions for their virulence. It may well be conceived, then, that a thorough knowledge of hygienic laws should form the basis of the education of every physician, and that some of the time of every head of a family might very profitably be spent in the reading of works on hygienic science. Those physicians who graduated some years since had not the opportunities for thorough acquaintance with the laws of health, and hence it is important that they should have at hand the best authorities upon the subject.

During the comparatively brief time since preventive medicine was raised to the dignity of a special branch of medical science, the field has become so extended that any work which claims to cover it all must of necessity give but cursory attention to certain departments, and that is the fault, if there be a fault, in the work under consideration. Yet it is sufficiently thorough to give one a perfectly clear idea of what is in accordance with, and what is in contravention of, a state of health. Some idea may be given of the comprehensiveness of it by an enumeration of the principal topics treated of in the book: The Air; Water; Food; Soil; Sewage; Dwellings; Hospitals; Schools; Occupations; Military, Camp, Marine, and Prison Hygiene; Exercise; Baths; Clothing; and the Disposal of the Dead. In addition there are chapters upon The Germ Theory of Disease; Contagion and Infection; History of Epidemic Diseases; Antiseptics and Disinfectants; Quarantine; and Vital Statistics.

The book is well written, and it contains a mass of information of the utmost importance, not only to every physician, but to all who desire to avoid illness and preserve the elasticity and strength that pertains to perfect health.

Basic Pathology and Specific Treatment of Diphtheria, Typhoid, Zymotic, Septic, Scorbutic, and Putrescent Diseases Generally. By Geo. J. Ziegler, M. D., late Physician to the Philadelphia Hospital; Author of "Zoo-Adynomia"; "Researches on Nitrous Oxide," etc., etc. Philadelphia: Geo. J. Ziegler, M. D., pp. 225. Price $2.00.

Dr. Ziegler is known in dentistry as one of the early editors of *The Dental Cosmos.* He is a ready and prolific writer, one who thinks for himself, and is not beholden to any one for his views on pathological subjects. He presents another consideration of the all engrossing medical topic of the present day, that of sepsis and antisepsis. The subject is yet too unsettled for the proper drawing of definite conclusions, and it behooves any one who would aim at intelligence to study it from more than one standpoint.

The author seems rather to ignore than to combat the later theories of Pasteur, Koch, and others concerning the origin of zymotic diseases, and to attribute them to chemical rather than fungoid origin. His theory is, that in all septic and infectious diseases, all of exanthematic and scorbutic disorders, necræmic tendencies, and putrid typhoid and dissolutive cases generally, the symptoms point to a general basic superalkaline condition of the blood and the system. This common morbific alkaline agent of these diseases he believes to be the well known ammonia, that is the constant product of the organic changes going on in the body, and he very ingeniously cites many well-known conditions and facts in support of his theory. The opponents of tobacco will find much to please them in the book, because the author declares that its use is one of the principal causes of the production of ammonia in the system. He believes in the spontaneous origin of many septic diseases, without the agency of micro-spores or germs, the favoring conditions requiring nothing but the presence of tobacco, with the products of decomposition.

The author has a wonderful vocabulary, and delights in multiplying names and technical terms. He is always positive and aggressive in his statements, sometimes indeed approaching dogmatism; but he is always earnest, consistent and congruous.

In his treatment of the diseases named he depends, as might readily be imagined, mainly upon acids.

The opponents of the germ theory of septic diseases will read the book with great delight.

Annals of Surgery, the new monthly journal, to be published simultaneously in this country and Great Britain, has made its appearance. The first number, of ninety-six pages, presents a handsome appearance, and contains some very interesting and instructive articles. With the very able editorial corps announced we may look for a journal of commanding influence.

$5.00 per annum. J. H. Chambers & Co., St. Louis.

Science. Who are subscribers to this journal? Every man who is interested in and earnestly desires to keep abreast the advance of general science. The journal is what its name indicates; it is science itself. Previous to its publication we had no journal so broad in its scope as is this. Since its first number it has gradually increased in interest, until now it is an essential to every man who would be thoroughly conversant with general scientific knowledge.

Cambridge, Mass. Weekly, $5.00 per annum.

The Sanatarian is one of the best journals that visits us. As its name indicates it is devoted especially to hygiene, and in its particular field it stands unrivaled. As preventive medicine assumes a position of greater importance, the necessity for such publications becomes more and more apparent, and the duty of physicians to keep pace with the advance of thought in this direction becomes an imperative one. It is ably edited by A. N. Bell, A. M., M. D., assisted by a distinguished corps of associates and collaborators.

Terms $4.00 per annum. Address 113 Fulton Street, New York.

Caulk's Dental Annual for 1884–1885. The Annual of last year contained a great mass of statistical and other information for dentists, and that for this year is nothing inferior. There is a list of the dental societies of the United States, with their officers, that alone is worth the price of the number to any who is interested in his profession. There are lists of dental colleges and periodicals, with digests of the various State dental laws, dental patents, number of dentists, dealers and dental manufacturers in the United States, with a large number of original articles and some judicious selections. We wonder what was the source of all the information contained, the most of which seems entirely reliable and accurate.

L. D. Caulk, D. D. S., Camden, Delaware. Price 25 cents.

Irregularities in Human Teeth, or Dental Teratology. By John J. R. Patrick, D. D. S , Belleville, Illinois. An Essay Read before the Illinois State Dental Society, at its Twentieth Annual Session, Springfield, N. Y., 1884.

Dr. Patrick has become well known as an authority in irregularities and malformations of the teeth and jaws. This pamphlet, of twenty pages, contains his latest ideas upon the subject, and is a thorough and systematic consideration of the causes and treatment of dental irregularity, that no one, unless like Dr. Patrick he has made an exhaustive study of his subject, is competent to prepare. We do not know that this pamphlet is for sale, but if it is not, it should be.

Transactions of the Odontological Society of Pennsylvania : From its Organization, Feb. 1st, 1879, to the Close of the year 1883.

From the secretary we have recevied the above-named volume. It is the record of the procedings of this important society for the first five years of its existence. It contains valuable papers from men like Guilford, Essig, Webb, Tees, Truman, Register, Bonwill, Kirk, and other members, with the discussions consequent upon their presentation. We could not say more for the contents if we were to write pages. The volume is handsomely bound, and is a credit to the society.

A few remaining copies are for sale by the secretary, Dr. Ambler Tees, 548 North 17th Street, Philadelphia. Price $3.00.

